# Supplementary material for: A Novel Tiled Amplicon Sequencing Assay Targeting the Tomato Brown Rugose Fruit Virus (ToBRFV) Genome Reveals Widespread Distribution in Municipal Wastewater Treatment Systems in the Province of Ontario, Canada
Source: Viruses. 2024 Mar 17;16(3):460. doi: 10.3390/v16030460 (PMC10974707; doi:10.3390/v16030460)
Supplement: Supplementary file 1 [file viruses-16-00460-s001.zip › Table_S1.pdf]

Table S1. List of sample sites, type of collection system, serviced population size, and raw data accessions.

Sample	Region	Collection Site	Collection System	Population	Sample Type	Collection Date	Prep. Date	RNA-Seq data accession	*ToBRFV*-Seq data accession
A	Kingston	Cataraqui Bay	Wastewater Treatment Plant	52 084	24-h composite	June 28th 2023	July 17th 2023	SAMN37915769	SAMN37915775
B	Peel	GE Booth	Wastewater Treatment Plant	1 089 738	24-h composite	July 10th 2023	July 17th 2023	SAMN37915770	SAMN37915776
C	Cambridge	Cambridge	Wastewater Treatment Plant	89 714	24-h composite	July 10th 2023	July 17th 2023	SAMN37915771	SAMN37915777
D	York	Warden - 407	Linear Pipe Collection Site	650 303	Grab	July 10th 2023	July 17th 2023	SAMN37915772	SAMN37915778
E	York	Leslie Street	Pumping Station	295 232	Grab	July 10th 2023	July 17th 2023	SAMN37915773	SAMN37915779
