# Supplementary material for: A Novel Tiled Amplicon Sequencing Assay Targeting the Tomato Brown Rugose Fruit Virus (ToBRFV) Genome Reveals Widespread Distribution in Municipal Wastewater Treatment Systems in the Province of Ontario, Canada
Source: Viruses. 2024 Mar 17;16(3):460. doi: 10.3390/v16030460 (PMC10974707; doi:10.3390/v16030460)
Supplement: Supplementary file 1 [file viruses-16-00460-s001.zip › Table_S2.pdf]

Table S2. Accession numbers of 179 ToBRFV Strains obtained from NCBI. ToBRFV genomes listed on NextStrain used to design primers and phylogenetic analysis. The strains used for tree building (PhyML Tree), assigned clades (Clade), and genomes used for read simulation (Sim. Reads) are listed. Unique strain genomes used in our PhyML tree are denoted yes (PhyML Tree), identical genomes are denoted no and the representative genome used in the PhyML Tree is listed (Rep. Genome). Strains were assigned to the same clade by both our PhyML tree and the NextStrain build, with the expectation of some clade 5 strains. Clade 5 strains predicted by NextStrain but not our analysis are labeled 5(N).

Accession	PhyML Tree	Rep. Genome	Clade	Sim. Reads
OM515230	yes	-	3	-
OM515231	yes	-	-	-
OM515232	yes	-	-	-
OM515233	yes	-	8	-
OM515234	yes	-	-	-
OM515235	yes	-	8	-
OM515236	yes	-	3	yes
OM515237	yes	-	-	-
OM515238	yes	-	3	-
OM515239	yes	-	1	yes
OM515240	yes	-	3	-
OM515241	yes	-	1	-
OM515242	yes	-	3	-
OM515243	no	MW314135	7	yes
OM515244	yes	-	3	-
OM515245	yes	-	7	-
OM515246	yes	-	7	-
OM515247	yes	-	7	-
OM515248	no	OM515255	7	-
OM515249	no	OM515252	7	-
OM515250	yes	-	-	-
OM515251	no	OM515252	7	-
OM515252	yes	-	7	-
OM515253	no	OM515255	7	-
OM515254	yes	-	7	-
OM515255	yes	-	7	-
OM515256	yes	-	-	-
OM515257	no	OM515266	-	-
OM515258	yes	-	8	yes
OM515259	yes	-	7	-
OM515260	no	OM515245	7	-
OM515261	yes	-	1	-
OM515262	yes	-	-	-
OM515263	yes	-	3	-

OM515264	yes	-	6	-
OM515265	yes	-	7	-
OM515266	yes	-	-	-
OM515267	yes	-	3	-
OM515268	no	OM515255	7	-
OM515269	no	OM515245	7	-
OM515270	yes	-	1	-
OM515271	yes	-	7	-
OM515272	yes	-	7	-
OM718702	yes	-	7	-
OM718703	yes	-	7	-
OM718704	yes	-	7	-
OM718705	no	OM515255	7	-
OM718706	yes	-	1	-
MZ945419	no	MZ945420	1	-
MZ945420	yes	-	1	-
MW349655	yes	-	4	-
MZ323110	yes	-	5	-
MZ438228	yes	-	5	-
MZ202349	yes	-	5 (N)	-
MZ004925	yes	-	5 (N)	-
MT018320	yes	-	5 (N)	-
MW314091	yes	-	5	-
MW314092	yes	-	2	yes
MW314093	no	MN882033	2	-
MW314094	yes	-	2	-
MW314095	no	MW314097	3	-
MW314096	no	MW314097	3	-
MW314097	yes	-	3	-
MW314098	no	MW314097	3	-
MW314099	no	MW314101	3	-
MW314100	no	MW314101	3	-
MW314101	yes	-	3	-
MW314102	no	MN882034	3	-
MW314103	yes	-	3	-
MW314104	no	MW314105	5	-
MW314105	yes	-	5	-
MW314106	yes	-	1	-
MW314107	no	MW314109	5	-
MW314108	yes	-	5	-
MW314109	yes	-	5	-
MW314110	yes	-	5	-
MW314111	yes	-	8	-
MW314112	yes	-	1	-
MW314113	yes	-	-	-
MW314114	yes	-	5	-
MW314115	no	MW314116	-	-

MW314116	yes	-	-	-
MW314117	yes	-	-	-
MW314118	yes	-	1	-
MW314119	no	MW314120	1	-
MW314120	yes	-	1	-
MW314121	yes	-	3	-
MW314122	yes	-	3	-
MW314123	yes	-	6	yes
KT383474	yes	-	5	-
MW314124	no	MW314125	1	-
MW314125	yes	-	1	-
MW314126	yes	-	3	-
MW314127	yes	-	3	-
MW314128	yes	-	1	-
MW314129	no	MW314136	3	-
MW314130	yes	-	1	-
MW314131	yes	-	1	-
MW314132	no	MW314137	1	-
MW314133	no	MW314135	7	-
MW314134	no	MW314135	7	-
MW314135	yes	-	7	-
MW314136	yes	-	3	-
MW314137	yes	-	1	-
MP872414	no	KT383474	5	-
MP872415	no	KX619418	5 (N)	-
MP875816	no	KT383474	5	-
MP875817	no	KX619418	5 (N)	-
MN549394	yes	-	4	yes
MN549395	yes	-	4	-
MN549396	yes	-	4	-
MT002973	yes	-	4	-
MT107885	yes	-	5 (N)	-
MT118666	no	KX619418	5 (N)	-
MN882011	yes	-	3	-
MN882012	yes	-	1	-
MN882013	yes	-	1	-
MN882014	yes	-	1	-
MN882015	yes	-	1	-
MN882016	yes	-	1	-
MN882017	yes	-	1	-
MN882018	yes	-	1	-
MN882019	no	MN882020	3	-
MN882020	yes	-	3	-
MN882021	yes	-	3	-
MN882022	no	MN882034	3	-
MN882023	no	MN882034	3	-
MN882024	no	MN882034	3	-

MN882025	no	MN882064	3	-
MN882026	no	MN882027	1	-
MN882027	yes	-	1	-
MN882028	no	MN882029	3	-
MN882029	yes	-	3	-
MN882030	no	MN882033	2	-
MN882031	no	MN882033	2	-
MN882032	no	MN882033	2	-
MN882033	yes	-	2	-
MN882034	yes	-	3	-
MN882035	no	MN882034	3	-
MN882036	yes	-	3	-
MN882037	no	MN882040	1	-
MN882038	no	MN882040	1	-
MN882039	no	MN882040	1	-
MN882040	yes	-	1	-
MN882041	yes	-	1	-
MN882042	no	MN882043	6	-
MN882043	yes	-	6	-
MN882044	no	MN882045	1	-
MN882045	yes	-	1	-
MN882046	no	MN882047	1	-
MN882047	yes	-	1	-
MN882048	yes	-	3	-
MN882049	no	MN882050	3	-
MN882050	yes	-	3	-
MN882051	yes	-	1	-
MN882052	yes	-	1	-
MN882053	yes	-	1	-
MN882054	yes	-	1	-
MN882055	yes	-	1	-
MN882056	no	MN882015	1	-
MN882057	yes	-	1	-
MN882058	yes	-	1	-
MN882059	no	MN882060	1	-
MN882060	yes	-	1	-
MN882061	no	MN882062	1	-
MN882062	yes	-	1	-
MN882063	no	MN882064	3	-
MN882064	yes	-	3	-
MN815773	yes	-	-	-
MN013187	yes	-	5 (N)	-
MN013188	yes	-	5 (N)	-
MN182533	yes	-	1	-
MK648157	yes	-	5 (N)	-
MN167466	yes	-	3 (5)	-
MK319944	yes	-	4	-

MK165457	yes	-	5 (N)	yes
MK133093	yes	-	5 (N)	-
MK133095	yes	-	3	-
KX619418	yes	-	5 (N)	-
