# Supplementary material for: A Novel Tiled Amplicon Sequencing Assay Targeting the Tomato Brown Rugose Fruit Virus (ToBRFV) Genome Reveals Widespread Distribution in Municipal Wastewater Treatment Systems in the Province of Ontario, Canada
Source: Viruses. 2024 Mar 17;16(3):460. doi: 10.3390/v16030460 (PMC10974707; doi:10.3390/v16030460)
Supplement: Supplementary file 1 [file viruses-16-00460-s001.zip › Table_S3.pdf]

Table S3. List of ToBRFV-Seq Primer Sequences and Concentration of Pooled Primers.

Primer Name	Pool	Sequence	Size	%GC	Tm	[Pool]
ToBRFV_1_LEFT	1	TTTACAACTACAATGGCATACACACA	26	34.62	59.95	0.5 µM
ToBRFV_1_RIGHT	1	ATTGGGCATACAGCAGTGAACA	22	45.45	60.74	0.5 µM
ToBRFV_2_LEFT	2	AATGATGCAGATCCCGTACGGA	22	50	61.79	0.5 µM
ToBRFV_2_RIGHT	2	CGGCGTAACAAACATGGACATT	22	45.45	60.27	0.5 µM
ToBRFV_3_LEFT	1	TGCTATTGCATTGCACAGTATATACG	26	38.46	60.56	0.5 µM
ToBRFV_3_RIGHT	1	TGCGCTGTAAAATTGCTCACTAT	23	39.13	59.56	0.5 µM
ToBRFV_4_LEFT	2	ACACCTGGTTTTGTAAGTTTTCTAGG	26	38.46	60.12	0.5 µM
ToBRFV_4_RIGHT	2	CTGGCAGTCACTCCGTTGATAA	22	50	60.53	0.5 µM
ToBRFV_5_LEFT	1	GCACTTATCAAGCCAAGGCACT	22	50	61.64	0.5 µM
ToBRFV_5_RIGHT	1	ATCAAGCACTGGCATATCCACC	22	50	60.93	0.5 µM
ToBRFV_6_LEFT	2	CGCATTAGAAATCAGGGTGCCT	22	50	61.19	0.5 µM
ToBRFV_6_RIGHT	2	CCTCTTGCCATTGAACCCTTCA	22	50	61	0.5 µM
ToBRFV_7_LEFT	1	TGTCGCATTGGCACTTAAAGATT	23	39.13	59.75	0.5 µM
ToBRFV_7_RIGHT	1	TGGTCGCAACATCTAAGACTCC	22	50	60.27	0.5 µM
ToBRFV_8_LEFT	2	CGGTGTCGAACCTAGTCAAGAT	22	50	60.02	0.5 µM
ToBRFV_8_RIGHT	2	CGGTACTAAGATTAGATCTTCCTCAAAAT	29	34.48	59.77	0.5 µM
ToBRFV_9_LEFT	1	GCAGTGCTAAAGTCGTCCTAGT	22	50	60.27	0.5 µM
ToBRFV_9_RIGHT	1	GAGTTTCCACCTCATCAACCTCT	23	47.83	60.25	0.5 µM
ToBRFV_10_LEFT	2	TGGAGACACACAACAAATTCCATAC	25	40	60.14	0.5 µM
ToBRFV_10_RIGHT	2	GCGATGATAGATACAGGTGTCGG	23	52.17	60.92	0.5 µM
ToBRFV_11_LEFT	1	ACGGACGTCCATACAGTACATG	22	50	60.08	0.5 µM
ToBRFV_11_RIGHT	1	CAGCATCATAGTTATTTAACATGGTGC	27	37.04	59.89	0.5 µM
ToBRFV_12_LEFT	2	GATGCAGGGACCCAATAGCAAT	22	50	60.94	0.5 µM
ToBRFV_12_RIGHT	2	TCTACTACCAAAGATGCAGTATTCTCA	27	37.04	60.15	0.5 µM
ToBRFV_13_LEFT	1	TTGGAAAATTTGGTGGCGATGA	22	40.91	59.55	0.5 µM
ToBRFV_13_RIGHT	1	TTTGCCTTGTGAGCTCACTGAA	22	45.45	60.87	0.5 µM
ToBRFV_14_LEFT	2	TTCAAAGCGAATATCCGGCCTT	22	45.45	60.86	0.5 µM
ToBRFV_14_RIGHT	2	TGAATGTTGTAACGTCCCCACT	22	45.45	60.08	0.5 µM
ToBRFV_15_LEFT	1	ACAAGGCCACAGGAAAACTACT	22	45.45	60.01	0.5 µM
ToBRFV_15_RIGHT	1	TGAACTCTTCTAAGTGATCCCAATCC	26	42.31	60.8	0.5 µM
ToBRFV_16_LEFT	2	ACGTGATACATCATGACAGAGGG	23	47.83	60.18	0.5 µM
ToBRFV_16_RIGHT	2	TGAACCAATATCTTATCAACCTTGGAGA	28	35.71	60.88	0.5 µM
ToBRFV_17_LEFT	1	TGAGTTCATAGACTTGTCAAAATCAGAA	28	32.14	59.87	0.5 µM
ToBRFV_17_RIGHT	1	CGCAGCAATTTTAACATTTCTAATATTAACGAG	33	30.3	62	0.5 µM
ToBRFV_18_LEFT	2	GGTTTCAGTTCAAAGTCGTTCCA	23	43.48	59.87	0.5 µM
ToBRFV_18_RIGHT	2	TCCTTCTCAACCCTAACTTTTAAACC	26	38.46	59.67	0.5 µM
ToBRFV_19_LEFT	1	ACCGGGAAAAAGTTTAGTAGTAAAAGTG	28	35.71	60.76	0.5 µM
ToBRFV_19_RIGHT	1	AGGATCTAGTACCGCATTGTACCT	24	45.83	61.02	0.5 µM
ToBRFV_20_LEFT	2	GGCCGACCCTATAGAATTAATAAATTTATGT	31	32.26	60.84	0.5 µM
ToBRFV_20_RIGHT	2	GGTGCAGAGGACCATTGTAAAC	22	50	59.69	0.5 µM
