# Supplementary material for: A Novel Tiled Amplicon Sequencing Assay Targeting the Tomato Brown Rugose Fruit Virus (ToBRFV) Genome Reveals Widespread Distribution in Municipal Wastewater Treatment Systems in the Province of Ontario, Canada
Source: Viruses. 2024 Mar 17;16(3):460. doi: 10.3390/v16030460 (PMC10974707; doi:10.3390/v16030460)
Supplement: Supplementary file 1 [file viruses-16-00460-s001.zip › Table_S4.pdf]

Table S4. Twenty-Six Virus Species common to all five wastewater influent shotgun samples.

Virus Species
Acanthamoeba polyphaga moumouvirus
Cactus virus X
Choristoneura fumiferana granulovirus
Cucumber green mottle mosaic virus
Emesvirus japonicum
Emesvirus zinderi
Enterobacteria phage GA
Enterobacteria phage Hgal1
Escherichia phage MS2
Garlic common latent virus
Garlic virus A
Hagavirus psychrophilum
Hubei picorna-like virus 61
Moumouvirus
Norwalk virus
Pepino mosaic virus
Pepper mild mottle virus
Pitaya virus X
Rehmannia mosaic virus
Schlumbergera virus X
Squash mosaic virus
Tobacco mild green mosaic virus
Tobacco mosaic virus
Tomato brown rugose fruit virus
Tomato mosaic virus
Wuhan insect virus 23
