# Supplementary figures and images for: A Novel Tiled Amplicon Sequencing Assay Targeting the Tomato Brown Rugose Fruit Virus (ToBRFV) Genome Reveals Widespread Distribution in Municipal Wastewater Treatment Systems in the Province of Ontario, Canada
Source: Viruses. 2024 Mar 17;16(3):460. doi: 10.3390/v16030460 (PMC10974707; doi:10.3390/v16030460)

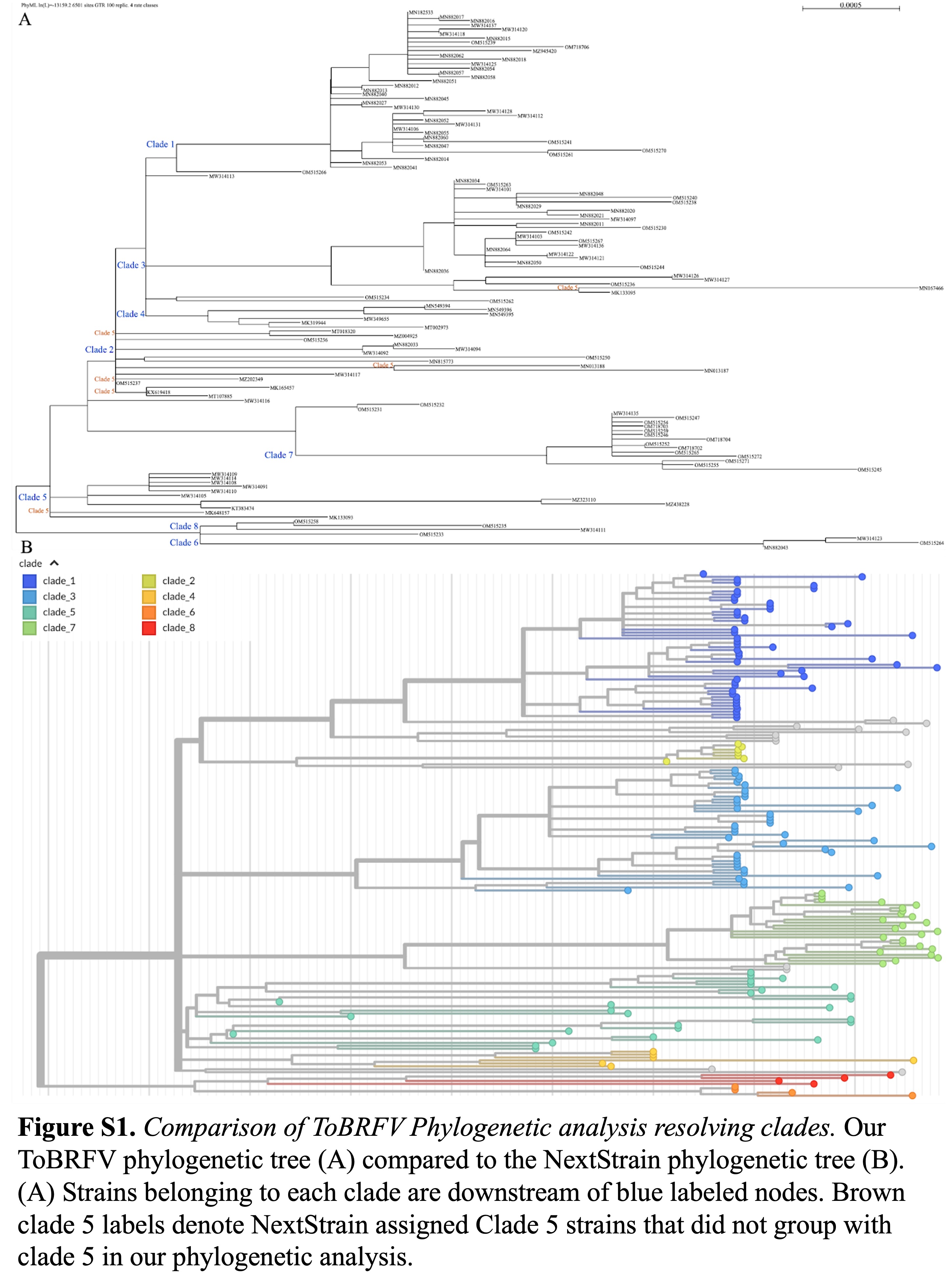

Supplement: Supplementary file 1 [file viruses-16-00460-s001.zip › Figure_S1.jpg]
